# COVID-19 and stroke in sub-Saharan Africa: case series from Dar es Salaam

**DOI:** 10.11604/pamj.supp.2020.35.24611

**Published:** 2020-07-03

**Authors:** Philip Babatunde Adebayo, Nadeem Kassam, Omar Aziz, Ahmed Jusabani, Samina Somji, Mugisha Clement Mazoko

**Affiliations:** 1 Neurology Section, Department of Medicine, Aga Khan Hospital, Dar es Salaam; 2 Department of Internal Medicine, Aga Khan University Dar es Salaam; 3 Department of Radiology, Aga Khan Hospital, Dar es Salaam; 4 Neurosurgery Section, Department of Surgery, Aga Khan Hospital, Dar es Salaam

**Keywords:** Stroke, COVID-19, neurology, Africa, Tanzania, case series

## Abstract

Low and middle-income countries including those in sub-Saharan (SSA) Africa are experiencing a steady increase in the number of COVID-19 cases. To the best of our knowledge, reports of COVID-19 related strokes are scarce in SSA. The peculiar situation of stroke care in SSA makes COVID-19 associated stroke a bothersome entity as it adds other dynamics that tilt the prognostic balance. We present a case series of COVID -19 related stroke in 3 patients from Tanzania. We emphasized protected code stroke protocol.

## Introduction

No doubt, SARS-CoV-2 infection and the coronavirus disease 2019 (COVID-19) is having a far reaching disruption of communities, countries and indeed, the global healthcare work force [1] Following the upsurge of cases outside China, in America as well as Europe, low and middle income countries (LMIC) including those in sub-Saharan Africa (SSA) are experiencing a steady increase in the number of COVID-19 cases. Health care institutions in SSA have not been insulated from the direct and collateral effect of the COVID 19 pandemic. 16th March 2020, Tanzania recorded the first case of COVID-19 [2]. Cases of the condition have since increased. Even though a rise in COVID-19 cases is being witnessed, they are in no way proportionate to the number of cases in the aforementioned continents. COVID-19 cases present with quite an array of systemic involvements [3]; including neurological features [4] but reports of COVID-19 related strokes are scarce in SSA. In this paper, we have presented 3 case reports of radiologically confirmed stroke cases in the setting of polymerase chain reaction (PCR)-confirmed SARS-CoV-2 infection.

## Methods

An audit of the patients with acute strokes who presented to the emergency department (ED) of the Aga Khan Hospital Dar es salaam (AKHD), Tanzania since April 1, 2020 was conducted. A chart review of patients with radiological confirmation of acute stroke and concomitant COVID-19 infection was performed.

## Results

**Figure 1:**
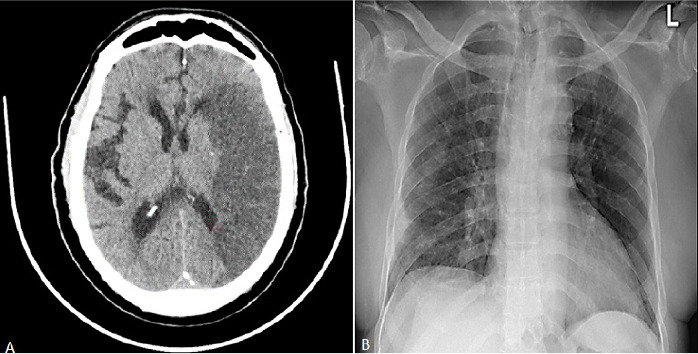
(A) non-contrast CT Head demonstrates left middle cerebral artery territory infarct with pressure effect effacing cortical sulci and ipsilateral lateral ventricle anterior horn; (B) AP chest X-ray revealed features of right lung pneumonia seen as patchy peripheral faint ground glass shadowing in the right lower zone

**Case 1:** 63-year-old male patient with background Diabetes mellitus (DM) and hypertension who was irregular on medication was brought to the emergency department (ED) with loss of power on his right side associated with progressive decline in mentation spanning 4 days. He had no previous transient ischemic attack. He did no smoke or drink alcoholic beverages. No cough, fever or respiratory symptoms were reported. No recent travel history or close contact with a sick person was established. On examination the patient´s consciousness was impaired, Glasgow coma scale (GCS) was 12 (best eye opening 4, best verbal 3, and best motor 5). On admission, the temperature was 36.70 C, pulse was 82 per minutes and blood pressure (BP) was 170/100 mmHg, pulse rate (PR) 82 b/min in normal sinus rhythm and was saturating well on room air at 97%. The admission NIHSS score was 26. Patient had right hemiparesis (power was 1/5 in the upper and lower limbs), down going planter response and depressed deep tendon reflex on the right side. He had no sign of meningeal irritation. On respiratory examination, he had coarse crepitations bilaterally. Other aspects of the cardiovascular and abdominal examination were essentially normal. A computed tomography (CT) confirmed left middle cerebral arterial infarct (Figure 1 A). A chest x-ray done on admission revealed patchy peripheral faint ground glass shadowing (Figure 1 B). Electrocardiogram (ECG) revealed left ventricular hypertrophy. At the ED, the patient received aspirin 75mg, clopidogrel 150mg, and atorvastatin 40 mg in addition to intravenous fluid.

He was thereafter admitted to the medical ward for continuity of care. Because of his x-ray findings, a possibility of COVID-19 was entertained hence; we commenced the hospital´s COVID- 19 protocol of management. We commenced him on intravenous ceftriaxone 1000mg twice a day, azithromycin 500 mg per- oral once a day, vitamin C 1000 mg per- oral twice a day, vitamin D 500 IU per- oral once a day, zinc 60 mg per- oral once a day (all via nasogastric tube). Subcutaneous heparin was also started at 5000 IU twice a day for thromboprophylaxis. His blood work up revealed significant leukocytosis with lymphopaenia, deranged renal function (Blood urea nitrogen, BUN was 26.16 mmol/l, Creatinine was 242.11 umol/l) and C-reactive protein was elevated to 153.03mg/l (Table 1).The patient´s condition grew progressively worse over a period of 24 hours. His GCS dropped to 10/15, he developed respiratory difficulty and worsening of kidney function. COVID-19 was later confirmed by nasopharyngeal and oropharyngeal swab using real-time reverse transcription PCR. The family was counseled on the prognosis and informed about mechanical ventilation and dialysis as the next step in management. However, the family chose not to escalate treatment and opted for comfort care. The Patient eventually passed away after few hours.

**Table 1:** Initial laboratory results of the cases

Haematological Parameters	Patient 1	Patient 2	Patient 3
White blood cell count (4.0 -11* 109/L	10.51	10.85	12.95
Neutrophils Absolute count (1.7-5.38)	9.22	7.77	10.60
Lymphocytes Absolute count (20.0-40.0)	0.67	18.2	1.68
Platelet count (150-450* 109/L)	406	177	349
Hemoglobin (14.0-17.5 g/dL)	12.8	15.5	10.9
Creatinine (59-104 umol/l)	242.11	128.76	136.78
BUN (2.76-8.07 mmol/l)	26.16	11.15	11.73
Prothrombin Time (control 11.8 seconds)	12.2	18.9	N/A
Activated partial thromboplastin (control 23.2 seconds)	23.2	24.3	N/A
D- Dimer (0-0.5 ug/ml)	**7.3**	**6.52**	**6.20**
Ferritin (22-322 ug/l)	1024	463	2601
Lactate Dehydrogenase (135-225 IU/L)	414	848	528.42
C- Reactive Protein serum (0.5-5 mg/L)	153	91.98	312
Procalcitonin	0.89	0.76	>100
Glycated hemoglobin (HB1Ac)	11.40%	N/A	10.24%
Total cholesterol (mmol/l)	3.39	5.50	2.69
Triglycerides (mmol/l)	1.60	1.27	2.45
HDL (>1.68 mmol/l No risk, 1.15-1.68 moderate risk, <1.15 high risk)	**0.78**	**0.80**	**0.46**
LDL (<optimal >2.59 mmol/l, Borderline 2.59 -3.34, Borderline high 3.37-4.12, high > 4.14)	1.88	4.22	1.12
VLDL	0.73	0.58	1.11

**Figure 2:**
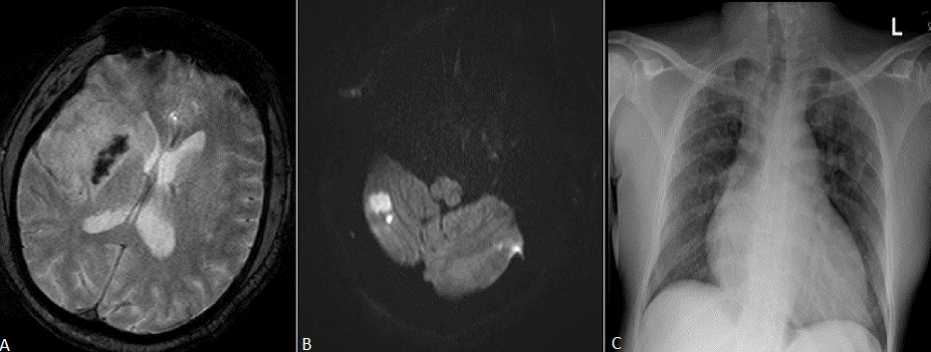
(A) GRE Haem sequence demonstrates low signal intensity in the region of right basal ganglia representing haemorrhagic transformation; (B) MRI DWI shows multiple hyperintense areas of restricted diffusion in the cerebellum in keeping with acute infarctions; (C): chest X-ray, AP reveals poorly defined infiltrates in the right upper zones suggestive of pneumonic changes

**Case 2:** a 38-year-old male recently diagnosed with heart failure, a month prior to admission, was brought to our ED with complaints of a weeklong history of acute left-sided weakness. He had also developed progressive altered mentation associated with fecal and urinary incontinence. Two days prior admission, he developed some degree of difficulty in breathing. On examination, he had a GCS of 9/15, NIHSS score was 28. His blood pressure was 109/56 mm Hg, pulse rate of 88 beats/min, oxygen saturation of 90% on room air, temperature of 37°C and a respiratory rate of 24 breaths/min. Auscultation of the chest revealed bilateral mild transmitted sounds. Patient´s brain magnetic resonance imaging (MRI) showed right temporal-parietal ischemic stroke with hemorrhagic transformation (Figure 2 A) as well as cerebellar infarcts (Figure 2 B). He was adjudged to have a cardio-embolic stroke in view of the multi-territorial distribution of the infarct. An x-ray of the chest revealed poorly defined infiltrates in the right upper zone suggestive of pneumonic changes (Figure 2 C). His laboratory tests are detailed in Table 1. A positive nitrite with 15-45 pus cells/hpf were noted in his urine analysis. The patient was admitted to the high-dependency unit of the isolation ward. He was supplemented with 10L/min of oxygen with a non-rebreather mask which resulted in improved saturation of 99%. He was started on intravenous ceftriaxone at 2000 mg once daily and metronidazole at a dose of 500 mg 8-hourly for possible aspiration pneumonia. Anti-platelets were held temporarily due to the hemorrhagic infarct. He was also started on oral Vitamin C 2000 mg once daily, Vitamin D 5000 IU once daily, Zinc 60 mg once daily via nasogastric tube. He was commenced on subcutaneous Enoxaparin 40 mg daily. A nasopharyngeal swab for SARS-Cov-2 PCR came back positive. Over a period of 72 hours the patient did not improve even with higher supplemental oxygen. The family opted for palliative care and no further tests were conducted. The patient succumbed a few hours after.

**Figure 3:**
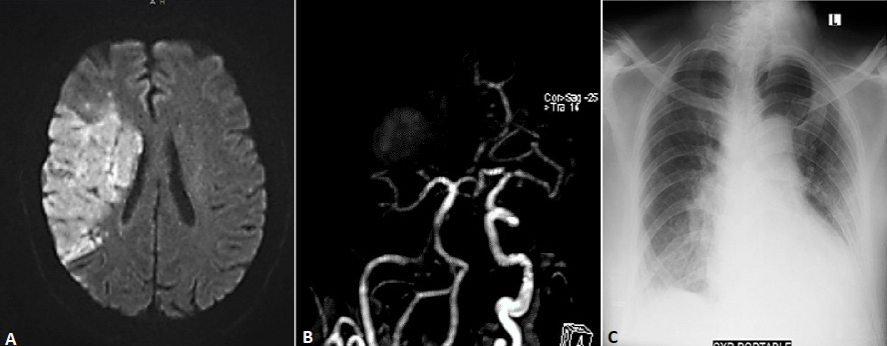
(A) MRI DWI revealed hyperintense signal in the right MCA territory which involved right fronto parietal temporal lobes as well as capsuloganglionic region, indicating abnormal fluid restriction in those areas; (B) 3D TOF MRA revealed extended right internal carotid (intracranial portion) stenosis with near-total occlusion of the right middle cerebral artery; (C) portable chest X-ray shows cardiomegaly and features of interstitial edema seen as bilateral parahilar and lower zone infiltrates

**Case 3:** this is a 63-year-old male patient known to have hypertension (>10 years) on nifedipine 20 mg twice a day. He was recently diagnosed with diabetes mellitus but irregular with the use of oral anti-diabetic medications. He was brought to the ED with a ten-day history of generalized body weakness associated with an intermittent non-productive cough. He had used some home remedies with no significant improvement. His clinical condition worsened with marked left sided body weakness and associated shortness of breath. Initial vital signs were significant for elevated blood pressure 170/100mmHg, with pulse rate of 94 b/min and regular. He was found to be desaturating to as low as 86% on room air. The Neurological examination was significant for central type facial weakness, depressed gag reflex, left hemiparesis (power 2/5) and left hypoaesthesia. His NIHSS score was 19. Respiratory examination was significant for bilateral crepitations from mid to lower zones, while cardiovascular examination revealed a displaced apex beat at 6th intercostal space, mid-clavicular line. The patient´s brain MRI and MRA revealed an acute right middle cerebral arterial infarct (Figure 3 A) and extensive right internal carotid artery stenosis (Figure 3 B) Laboratory work up (Table 1) was significant for neutrophylic leucocytosis, increased ferritin and lactate dehydrogenase. An x-ray of the chest (Figure 3 C) was suggestive of interstitial edema, while echocardiography on admission revealed features suggestive of left ventricular hypertrophy and global hypokinesia with ejection fraction of 40%. A nasopharyngeal swab for SARS-Cov-2 PCR was taken on admission, which came back positive.

The patient was admitted to the high-dependency unit of the isolation ward and was supplemented with 4L/min of oxygen with nasal prong to maintain saturation above 95%. He was started on intravenous Ceftriaxone at the dose of 1000 mg twice daily, oral Vitamin C 2000 mg once daily, Vitamin D 5000 IU once daily, Zinc 60 mg once daily and subcutaneous Enoxaparin 40 mg daily. In addition, daily bisoprolol 2.5 mg, losartan 50 mg, frusemide 40 mg, and eplerenone 25 mg were commenced on account of his heart failure. Patient´s blood sugar was well controlled with subcutaneous Insulin glargine 20 IU and sitagliptin 100 mg once a day. The patient improved over the course of admission; maintaining saturation of above 95% on room air, with good glycaemic and blood pressure control. He was subsequently discharged from the general ward 72 hours later for self-isolation. A repeat nasopharyngeal swab for SARS-Cov-2 PCR 14 days after discharge was negative.

## Discussion

We have reported 3 patients with COVID-19 associated ischaemic strokes. To the best of our knowledge, this the first series of COVID-19 related stroke in SSA. It is already known that the clinical course of COVID-19 is most severe among the elderly, the male gender, and patients with underlying conditions; particularly diabetes, hypertension and metabolic syndrome [5]. Our patients were all males (2 elderlies and 1 young man) who had comorbid conditions (diabetes, hypertension, heart failure). Although previous reports suggest that the average time of onset of stroke after COVID-19 diagnosis was 12 days, [6] two cases actually had stroke as their first diagnoses in our series. Indeed, the first case reported no cough. However, his progressive decline and desaturation in the intensive care informed the clinical decision to test for COVID-19; he had only been ill for 4 days. Elevated D-dimer, low HDL and late presentation stand out as denominators for these patients. Late presentation of stroke cases to the ED is commonplace in SSA prior to the pandemic. However, there is a recent change in the health seeking behaviour during this era. This could be related to a smaller proportion of patients seeking healthcare services for milder symptoms [7]. The “stay at home” mantra appears to influence this.

The interaction between infections and stroke has been well established [8]. Systemic infections may cause ischaemic or haemorrhagic stroke by direct vascular invasion, thrombosis, or vasculitis. Viral respiratory infections may increase the susceptibility to stroke by inducing a systemic inflammatory response that can lead to a hypercoagulable state, destabilization of pre-existing atherosclerotic plaques, and local thrombosis [8] In COVID-19 patients, endothelium damage mimicking vasculitis could be seen in severe cases [9] In limited autopsy samples, congestions of alveolar septal blood vessels and lymphocytes/monocytes infiltration within and around blood vessels have been described. In addition, small blood vessels did show hyperplasia, vessel wall thickening, lumen stenosis, occlusion and focal haemorrhage. A proportion of the severe cases had hyaline thrombi of micro-vessels [10,11]. Hypercoagulable state (elevated antiphospholipid antibodies) [12] and other perturbations of the coagulation system (abnormal platelets and D-dimer) [13], including elevated inflammatory biomarkers [13] are all possible underlying mechanism for increased risk of stroke as well as worsening of symptoms in COVID-19.

Although our hospital is stroke ready, these cases came outside the therapeutic window period hence they were not triaged to the hyperacute stroke pathway. The management of these patients were directed at preventing secondary brain injury. Although current data suggest use of prophylactic anticoagulation with low molecular weight heparin for COVID-19 inpatients, [14] these agents pose a risk of haemorrhagic transformation of ischaemic stroke hence treatment should be individualized. Neuro-critical care of stroke patients sometimes required mechanical ventilation. In patients with significant respiratory illness or failure, timely mechanical ventilation improves stroke outcome [15]. However, in SSA with limited resources, this might not be always feasible. Our first patient was offered the service, but the relatives declined. Certainly, COVID-19 adds another dimension to the already existing poor outlook of acute stroke care in SSA. More worrisome is the risk of infections to members of the stroke team thereby shrinking the already modest number of stroke professionals [16]. In our hospital, two members of our team fell ill during this period leading to a serious strain on the remaining stroke team members. Protected code stroke (PCS) initiative has been proposed to prevent this risk [17]. The key components of the protocol include infection control screening, use of droplets and contact personal protective equipment (PPE) and crisis resource management. The population must need also be continuously educated and reminded on the benefits of early hospital presentation of stroke cases.

## Conclusion

While stroke remains a medical emergency requiring swift medical and surgical intervention, concurrent COVID-19 infection presents a new challenge that tilts the prognostic balance. Furthermore, the implications for stroke team and stroke ready hospitals in SSA have been highlighted. An effort at maintaining universal precautions and donning of appropriate PPE for every suspected patient is advised but we must not lose precious time on this endeavour. Time is still brain.

### What is known about this topic

- COVID-19 associated neurological disorders including strokes are being reported;
- Most cases are reported outside sub-Saharan Africa;
- Many protocol to enhance prompt treatment and limit healthcare professional exposure are developed.

### What this study adds

- The case series emphasized the peculiar challenges of stroke care during COVID-19 era in sub-Saharan Africa;
- Both haemorrhagic and ischaemic strokes are possible in the setting of COVID-19;
- Protected Code Stroke is emphasized to limit healthcare professional exposure.

## Competing interests

The authors declare no competing interests

## Authors’ contributions

PBA and NMK conceived the idea of the manuscript. PBA, NMK, OMA drafted the manuscript. AJ reviewed the manuscript and imaging reports. SSS and MCM reviewed and edited the manuscript for intellectual content.
